# Transition from Enteral to Oral Nutrition in Intensive Care and Post Intensive Care Patients: A Scoping Review

**DOI:** 10.3390/nu17111780

**Published:** 2025-05-24

**Authors:** Gioia Vinci, Nataliia Yakovenko, Elisabeth De Waele, Reto Stocker

**Affiliations:** 1 Department of Clinical Nutrition, Klinik Hirslanden Zurich, 8032 Zurich, Switzerland; 2 Department of Intensive Care Medicine, Klinik Hirslanden Zurich, 8032 Zurich, Switzerland; 3 Faculty of Medicine, University of Zurich, 8006 Zurich, Switzerland; 4 Department of Clinical Nutrition, Klinik Im Park Zurich, 8027 Zurich, Switzerland; nataliia.yakovenko.zrh@gmail.com; 5 Metabolism & Nutrition (MENU) Research Unit, Vitality Research Group, Vrije Universiteit Brussel (VUB), Laarbeeklaan 103, B-1090 Brussels, Belgium; elisabeth.dewaele@uzbrussel.be; 6 Clinical Nutrition Department, Vrije Universiteit Brussel (VUB), Universitair Ziekenhuis Brussel (UZB), Laarbeeklaan 101, B-1090 Brussels, Belgium; 7 Department of Teaching & Research, Klinik Hirslanden Zurich, 8032 Zurich, Switzerland; reto.stocker@hirslanden.ch

**Keywords:** enteral nutrition, oral nutrition, intensive care medicine, critical care, feeding tube, transition process

## Abstract

**Background:** Limited information exists regarding the current practice of transitioning from enteral nutrition (EN) to oral nutrition (ON) and the effect of this process on the relationship between energy and protein requirement, provision, and nutritional status of intensive care and post-intensive care patients. Current practices and policies to the transition from EN to ON based on perspectives, experiences and opinions of health professionals and patients, are neither widely understood nor consistently implemented. **Aim:** The scoping review aims to summarize the current state of research on the transition process from EN to ON in intensive care unit (ICU) patients and post-ICU patients. The aim is to understand the impact of this process on the relationship between energy and protein requirements, and provision, as well as the impact on nutritional status. Additionally, the review aims to gather insights into the perspectives, experiences and opinions of healthcare professionals and patients regarding the transition process and the removal of enteral feeding tubes. **Design:** The literature search was conducted in PubMed, Cochrane Library and Scopus. Keywords and MeSH terms were applied, with additional papers identified by snowballing. Publications were manually screened based on inclusion and exclusion criteria to determine eligibility for inclusion. **Results:** A total of six studies were identified on this topic. One study found that, after the feeding tube was removed after ICU discharge, energy intake decreased from 97.3% to 65% and protein intake decreased from 91.5% to 60.6% of target values within one day after removal. Five additional studies revealed that the removal of feeding tubes is often a primary goal for nurses and physicians on the ward, and the decision to remove the tube is not based on an assessment of potential oral energy and protein intake. Reinsertion of a feeding tube is viewed as a setback by nurses and physicians. The process and decision-making of the tube removal seems to be unclear as well as the involvement of patients in the process. No studies were found examining the correlation between nutritional status and the transition process. **Conclusions:** Energy and protein intake appear to decrease directly after removal of the feeding tube. The decision to remove a feeding tube is often influenced by the personal opinion of healthcare professionals or institutional practices, rather than on the basis of an assessment of oral energy and protein intake. Additional studies are needed to further explore the transition process, the perspectives and experiences of healthcare professionals, and the impact of the process on energy and protein adequacy as well as the nutritional status of ICU and post-ICU patients.

## 1. Introduction

On admission to the intensive care unit (ICU), 38–78% of patients are malnourished [1]. Persistent malnutrition and inadequate energy and protein adequacy in the ICU are associated with an increased risk of nosocomial infections, longer antibiotic therapy, longer ventilation times, prolonged hospital stays, higher readmission rates and increased mortality [1,2,3,4,5,6]. To ensure adequate nutrition in the ICU, oral nutrition (ON), enteral nutrition (EN) and/or parenteral nutrition (PN) routes are used. International nutrition guidelines recommend ON over EN or PN in ICU patients who are able to eat and when ON is not feasible, EN should be prioritized over PN [7,8,9].

Data indicate that only 39% of ICU patients are able to eat orally [10] and oral intake in the ICU is generally low [11,12,13,14,15,16]. The use of ON as a feeding route in the ICU is limited due to factors such as inappetence, early satiety, nausea, vomiting, swallowing disorders, dysgeusia, delirium, and weakness [11,12,14,15,17]. Dysphagia is particularly common after extubation [18,19,20,21,22]. Even 7 days post-extubation, oral intake remains inadequate, with energy adequacy reaching only <47–55% and protein adequacy < 27–34% of target values [14,15]. Because ON is often not a viable nutritional option, particularly during the early acute phase, international guidelines recommend initiating supportive nutrition through EN and/or PN at an early stage [7,8,9]. In the ICU, 49% of patients receive EN [10] with a mean duration of 15.2 ± 28.9 days [23], while only 24% of patients receive PN [10]. Data from the worldwide nutrition day (2007–2021) shows that EN via feeding tubes is a commonly used and important nutritional approach for this patient group [10].

As patients progress through their hospital stay, particularly toward the end of their ICU stay or following ICU discharge, a transition from EN to ON typically occurs, during which EN is stopped and feeding tubes are removed. Enteral feeding tubes are often removed still in the ICU or shortly after ICU discharge on the ward [12,24]. However, many symptoms that hinder ON in the ICU, such as nausea/vomiting, dysphagia, loss of appetite, and altered consciousness [25] often persist after discharge, making it highly likely that patients on the ward will struggle to eat sufficient oral nutrition and may still require the feeding tube. The literature provides limited recommendations against premature removal of feeding tubes [12,24]. International guidelines do not offer any specific recommendation on the transition process of the optimal timing for feeding tube removal [7,8,9]. Potential benefits and harms of this transition process are outlined in Table 1.

Little is known about current practices surrounding the transition from EN to ON and the correlation of this process on the energy and protein adequacy and nutritional status of ICU and post-ICU patients. The research team hypothesizes that feeding tubes are often removed too early in post-ICU patients, leading to inadequate energy and protein adequacy and negatively impacting nutritional status. They also believe that while the decision-making process for removing feeding tubes during this transition is important, it remains poorly understood.

The primary aim of this scoping review is to summarize the existing research on the transition from EN to ON in ICU and post-ICU patients, with a focus on its impact on energy and protein adequacy and nutritional status. The secondary aim is to explore and synthesize the views, experiences, and perspectives of healthcare professionals and patients regarding the decision-making process of enteral feeding tube removal.

## 2. Materials and Methods

A scoping review was conducted in January and February 2025 to systematically present the research in this area and to identify gaps in knowledge. The PRISMA guidelines for scoping reviews were followed to ensure high-quality standards [26]. The following research questions guided the review:

1. What is known from the literature about energy and protein adequacy in ICU and post-ICU patients during the transition process from enteral to oral nutrition?
2. How does the nutritional status of the patients correlate with this transition process?
3. How do patients and healthcare professionals experience the transition process including the decision-making process of feeding tube removal?

To be included in the scoping review, studies published in medical journals needed to focus on the transition from EN to ON in adult ICU and post-ICU patients (aged > 18 years). Studies had to focus on the nutritional status and/or energy and protein adequacy before and after feeding tube removal and/or explore the experiences of patients or healthcare professional with the transition process. Quantitative, qualitative and mixed-method studies were considered to capture diverse perspectives on the topic.

Exclusion criteria included studies that did not focus on the transition process, studies involving children (aged < 18 years), and studies published in languages other than German or English.

**Figure 1:**
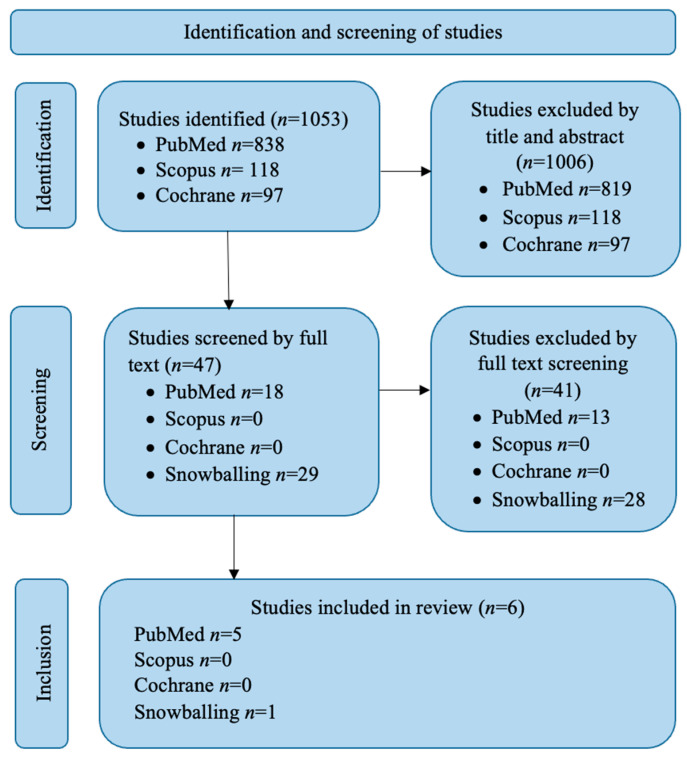
Chart for study identification, screening, and inclusion.

Relevant studies were identified by searching PubMed, Cochrane Library and Scopus using keywords such as ‘enteral nutrition’, ‘feeding tube’, ‘nutrition status’, ‘nutrition assessment’, ‘energy’, ‘protein’, ‘critical illness’, ‘intensive care unit’, ‘intensive care’, ‘transition’. MeSH terms were also employed to ensure a comprehensive search. No restriction were applied regarding publication dates. Additional studies were identified through snowballing by screening the reference lists of the included studies. The detailed search strategy for PubMed is provided in Table A1 in the Appendix A of this review. The first and second authors independently evaluated studies by reviewing titles and abstracts, following by full-text screening to identify relevant publications. Any discrepancies were resolved through discussion. A flowchart detailing the study identification, screening and inclusion process is presented in Figure 1.

## 3. Results

During the search process, a total of 1053 articles were identified (after removal of duplicates) and screened based on their titles and abstracts. In total 47 studies (including those identified through snowballing) underwent full-text screening to assess their eligibility. Ultimately, six studies were included in the scoping review. One study examined energy and protein adequacy in relation to the transition process, while five studies explored the opinions, experiences and perspectives of healthcare professionals regarding the decision-making process for removing enteral feeding tube in practice. No studies were identified that addressed the correlation between nutritional status and the transition process or the experiences of patients during the transition. The included studies were published between 2013 and 2024, with most appearing in Pubmed. Table 2 provides an overview of the included studies, detailing the authors, title, study designs, publication years and the key findings regarding the research question of this scoping review.

### 3.1. Energy and Protein Adequacy in Correlation with Transition Process

Only one study was found that investigated the effects of transition from EN to ON energy and protein adequacy in ICU and post-ICU patients.

The prospective observational single-center cohort study (PROSPECT-1) by Slingerland-Boot et al. [27] primarily aimed to assess energy and protein adequacy among patients receiving oral, enteral, parenteral or combined nutrition after ICU discharge. The study recruited adult ICU patients discharged to the ward after an ICU stay of ≥72 h, during which they received EN for ≥24 h. The study took place in a mixed medical-surgical ICU in a hospital in the Netherlands. Patients exclusively on ON, with a life-expectancy of less than 48 h, or not discharged to the ward were excluded. A total of 41 patients were included in the analysis, with a mean age 70.8 years (SD 11.4), mean BMI 26.7 (SD 6.0), APACHE II score at ICU admission of 20.4 (SD 6.7), and a SOFA score at ICU admission of 6.6 (SD 2.8). Swallowing function at ICU discharge was good in 68.3%, moderate at 24.4% and bad in 7.3%. Additionally, 56% of participants were non-surgical patients.

The results of the study suggest a decline in nutritional intake following feeding tube removal in the initial days after ICU discharge. Median energy adequacy decreased from 97.3% to 76% and median protein adequacy dropped from 91.5% to 75.4% of target values. The most significant decline occurred on the first day after feeding tube removal, with median energy adequacy falling to 65% and a median protein adequacy to 60.6%. Supportive EN was provided for a median duration of three days before tube removal. Patients exclusively receiving ON exhibited the lowest nutritional intake after ICU, whereas those with EN support had the highest adequacy. However, the study did not provide detailed information about the type of oral diet used before and after feeding tube removal (e.g., texture-modified or energy- and protein-enriched diets). The authors noted that premature feeding tube removal poses a risk to nutritional status. The study faced limitations due to its small sample size (*n* = 41) and recruitment had to be stopped early because of COVID-19 pandemic.

### 3.2. Experience and Decision-Making of Transition and Feeding Tube Removal

Five studies were found that explored the experiences of healthcare professionals (nurses and physicians) during the transition from EN to ON and the decision-making process for removing feeding tubes. Only one study addressed the perspectives of post-ICU patients, focusing on barriers to ON rather than the transition process itself.

Chapple et al. [28] conducted a qualitative study using semi-structured interviews with ICU and ward nurses and physicians (*n* = 34). The study highlighted that early feeding tube removal in ICU and post-ICU patients with traumatic brain injury was a primary goal for both nurses (*n* = 18) and physicians (*n* = 16). Feeding tube reinsertion was perceived as a setback. Nurses and physicians of the ward prioritized helping patients to normalcy quickly (getting back to normal), tolerating weight loss during recovery. Additionally, the study revealed that nutrition was rarely discussed during ward rounds.

Merriweather et al. [29] conducted a grounded theory approach, including meal observations, gathering information relating to nutritional intake on the ward and semi-structured interviews with post-ICU patients (*n* = 17) with a history of more than two days of mechanical ventilation during ICU. The aim of the study was to investigate nutritional problems of this patient group and to examine the organizational issues that influence nutritional care after discharge from ICU. Merriweather et al. saw a general ward ethos of removing any lines including feeding tubes as soon as possible after ICU discharge. The feeding tube removal is often performed without formal assessment of energy and protein intake by a dietitian and out of hours when no dietitian was present to enable an estimate of the oral coverage of energy and protein requirements. The observation showed a minimal ON intake after feeding tube removal. One reason for early feeding tube removal was seen in a lack of nutritional knowledge among ward healthcare professionals about nutritional needs of post ICU patients. Patients mentioned different barriers with ON as, e.g., issues with mealtimes and timing. Authors recommend avoiding the removal of feeding tubes at or near the time of extubation. Furthermore, they recommend an education of healthcare professionals (nurses, dietitians and physicians) of the ward about the special nutritional needs and problems of post-ICU patients.

A lack of knowledge resulting in the removal of feeding tubes, often directly after extubating or shortly after ICU discharge, was described, next to Merriweather et al., also in a narrative review by Moisey et al. [30]. Nutrition care plans seem to be poorly communicated between healthcare professionals and nutrition appears to be seen as low priority.

Zaher et al. [31] conducted a cross-sectional study using an online survey. The survey asked ICU nurses (*n* = 136) to rate 24 potential barriers to EN based on their perceived importance. Results revealed that feeding tubes were often removed without clear documentation or an explicit physician’s prescription. This lack of standardization highlighted the variability in feeding tube management practices.

ApSimon et al. [24] performed a retrospective, single-center study in a 30-bed ICU in Canada. Patients included in the study were ≥18 years old, required mechanical ventilation during their ICU stay, and received EN for a minimum of three days before being discharged to the ward. Patients were excluded if they received PN after ICU discharge, had limited life expectancy due to palliative care, or were discharged outside of the study hospital. The ICU included neurological, trauma, medical and surgical patients. The study analyzed 63 patients, with a mean age of 60.5 years (SD 15.3), a mean BMI of 26.5 (SD 5.9), and a mean APACHE II score of 21.0 (SD 7.4). Admission diagnosis included respiratory illness (25.5%), neurological disease (18.6%), trauma (14.7%), sepsis (12.7%) and others (63.4%). Inotropes/vasopressors were used in 63.4% of cases. Among the patients, 55.9% were discharged from the ICU without feeding tube and in 71% of post-ICU patients receiving EN, feeding tubes were removed from the ward due to hospital policies rather than because energy and protein needs were met orally. In 18.4% of the patients the feeding tube was removed before ICU discharge. The study found that the longer patients remained in the ICU, the more likely it was that the feeding tube remained in place even after transferring to the ward. Patients who did not receive EN were at higher risk of unplanned hospital readmission. The study did not provide information about the type of oral diet administered before or after tube removal. The authors recommended that feeding tubes should only be removed based on an individual’s ability to meet oral energy and protein requirements, rather than following institutional practices. They also emphasized the importance of educating healthcare staff about the risks of inadequate oral nutrition and the consequences of premature feeding tube removal.

### 3.3. Nutritional Status in Correlation with Transition Process

No studies were found investigating the nutritional status of ICU and post-ICU patients in correlation with the transition from EN to ON.

## 4. Discussion

The studies reviewed in this scoping review suggest that energy and protein adequacy often decline after feeding tube removal in ICU and post-ICU patients. Ensuring adequate ON in the ward, following tube removal, appears to be challenging. As a result, several authors recommend delaying feeding tube removal in this vulnerable patient population at risk of malnutrition. Additionally, research indicates that feeding tubes are frequently removed prematurely by nurses and physicians without a thorough assessment of the patient’s oral feeding situation or a consultation with a clinical dietitian. The scoping review was conducted to lay the foundation for future research on the transition from enteral to oral nutrition in ICU and post-ICU patients, using the current evidence available.

### 4.1. Oral Nutrition After ICU Discharge

Slingerlang-Boot et al. [27] demonstrated a decrease in energy and protein adequacy with only ON after cessation of EN and removal of feeding tubes. This appears to be the only study so far focusing on energy and protein adequacy in the context of the transition process. This leads to a research gap in what actually happens with respect to the ON and EN in these vulnerable patients after discharge from the ICU. Premature feeding tube removal may exacerbate or contribute to malnutrition, yet limited data exist to fully quantify this risk or address the underlying issues.

There is more literature available on the general oral energy and protein adequacy of post ICU patients without a focus on transition. A systematic review by Rosseel et al. [32] found that post-ICU patients receiving EN or a combination of ON and EN achieved the best energy and protein adequacy. The studies included in the review reported an energy adequacy between 52 and 102% and a protein adequacy between 63 and 83% of target values. The removal of the feeding tube and a poor recording of ON intake by staff were cited as barriers to adequate nutrition in this patient group. Immediately after ICU discharge, patients had low energy (47%) and protein (27%) adequacy with the lowest adequacy observed in patients relying solely on ON [14]. Knudsen et al. [33] observed a significant decline in energy adequacy from 94% to 30.5% (*p* = 0.0051) and protein adequacy from 73% to 27.5% (*p* = 0.0117) within three days of ICU discharge. Jarden et al. [12] found that 62% of post-ICU patients consumed less than 2/3 of their meals, and 66% consumed less than 1/3, often due to low appetite and early satiety. Yatabe et al. [16] reported an oral energy intake of only 10.8 kcal/kg/day and an oral protein intake of only 0.4 g/kg/day after ICU discharge. Fischer et al. [34] confirmed a low oral intake in this population. In addition, Chapple et al. [35] and Rousseau et al. [36] showed a reduced oral intake even twelve months after ICU discharge, with oral energy adequacy of 82.7% and protein adequacy of 71.7%, largely due to persisting loss of appetite and dysphagia. These findings underscore the critical need for improved nutritional management and support during and after the transition from ICU care.

### 4.2. Energy and Protein Requirements After ICU

At this point, it makes sense to look at energy and protein requirements after ICU, although only a few recommendations can currently be found. Van Zanten et al. [37] recommend 30 kcal/kgBW and 1.5–2.0 g/kgBW protein during the post-ICU phase and 35 kcal/kgBW and 2.0–2.5 g/kgBW Protein after hospital discharge. Some studies show a measured energy expenditure (by indirect calorimetry) of 23.1 (20.9–24.4) kcal/kg/day for female and 22.6 (18.6–24.4) kcal/kg/day for male post-ICU patients [38]. The research group by Rosseel highlighted the different methods used to calculate nutritional targets and report results in studies on post-ICU patients. Energy needs of post-ICU patients in the studies are calculated, for example, with the Schofield equation, 25 kcal/kgBodyweight (BW), FAO/WHO Equation 25–30 kcal/kgBW or measured by using indirect calorimetry [32]. Also, the calculation of protein needs varies considerably between 0.8 and 2.0 g/kgBW for the calculations. For the reporting of oral intake 24 h recalls, food charts or weighed methods are used. However, non-nutritional calorie sources, such as glucose infusions, propofol or ethanol were not consistently included in energy calculations. This fact complicates the comparison of nutritional adequacies across studies involving ICU and post-ICU patients.

### 4.3. Nutritional Status of ICU and Post-ICU Patients

No studies were found looking at the evolution of the nutritional status of ICU and post-ICU patients in correlation with the transition from EN to ON. Although no studies could be found, several studies show a change in the nutritional status of patients in the ICU. Especially in muscle mass during ICU stay and also after ICU discharge. Studies showed significant weight loss in ICU from 3.9% [39], to 11.3% [20], and up to 22.5% [40] of pre-ICU weight during ICU stay. Nematy et al. [17] showed a significant decrease in weight from ICU admission to ICU discharge (78.4 ± 0.7 vs. 68.1 ± 5.2 kg, *p* = 0.03). Next to weight loss also the mid-upper arm circumference seemed to decrease during ICU stay with a change from 33.2 to 29.3 cm from ICU admission to discharge [11] and a medium loss of 1.6% per day [41]. Calf circumference changed from 39.5 to 37.5 cm [11] or from 31.4 ± 4.2 cm at baseline to 30.2 ± 4.0 cm at discharge (*p* < 0.001) [42]. In addition, studies showed a reduction in quadriceps muscle layer thickness of 7.2% (−0.23 cm) after ICU discharge [39] and from 2.6 to 2.5 cm from ICU admission to ICU discharge [11]. Parry et al. [43] showed a reduction of 30% of quadriceps thickness within the first 10 days of ICU and Puthucheary et al. [44] a reduction of 17.7%.

One aim of this scoping review was to explore the impact of the transition on nutritional status. No studies were found that addressed this correlation. Further research is needed to understand how the transition process affects the nutritional status and therefore the nutritional outcomes of patients after ICU.

### 4.4. Patients’ Perspective on Oral Nutrition After ICU

Segaran et al. [45] explored patient’s perspectives on initiating ON after ICU discharge. Their study revealed that starting ON after a period of being nil by mouth in the ICU was often viewed as a positive sign of recovery, instilling hope and optimism. However, it was also associated with frustration and anxiety [45]. Research has shown that factors such as loss of appetite, early satiety, taste alterations, fatigue, anxiety, low mood, and lack of motivation negatively impact oral intake in post-ICU patients [30,46,47]. Moisey et al. [30] further identified additional barriers, including nausea, vomiting, dysphagia, ICU acquired weakness, depression, post-traumatic stress disorder, dysmorphia, pain, fatigue, and many other factors. Up to 62% of post-ICU patients experience dysphagia after ICU discharge, along with loss of appetite and taste change [48]. Hardy et al. [49], in their narrative review, provided a comprehensive overview of the patients’ experiences with nutritional therapy during the ICU and the recovery phase. This information appears to be important in addition to the discussion about nutritional situations, energy and protein intake. Since the issue of inadequate energy and protein intake after ICU discharge remains poorly understood, future studies should explore patient conditions in greater depth, including factors such as consciousness levels, vital signs, medications, electrolyte imbalances, bowel function, pre-existing maldigestion or malabsorption, ventilation modifications, and the type and duration of anesthesia.

### 4.5. Experience of Transition Process and Decision-Making

International nutritional guidelines generally serve as a framework to guide decision-making in clinical nutrition therapy. However, no specific recommendations exist for the transition from EN to ON in ICU and post-ICU patients. As a result, practical decisions in this area are often based on principles that are not yet well understood. This scoping review identified only a few qualitative studies that examined the opinions, experiences and decision-making processes related to feeding tube removal in practice. Merriweather et al. [29] and Chapple et al. [28] highlighted a strong inclination among nurses and physicians to remove any lines, including feeding tubes, as soon as possible after ICU discharge. The underlying reasons for this urgency and the phenomenon of premature tube removal remains insufficiently explored. Furthermore, the perspectives of the various professional groups involved in these decisions have not been thoroughly analyzed. For instance, dietitians, who frequently manage post-ICU patients on the ward and are uniquely qualified to assess oral nutrition adequacy and advise on the optimal timing for feeding tube removal, were not consulted in these studies. This oversight leaves important questions about their insights and contributions unanswered.

In addition to nurses, physicians and dietitians, patients should also be involved in this decision-making process regarding feeding tube removal. It is crucial to know patients’ opinions, experiences and thoughts about EN and feeding tubes. Hazzard et al. [50] conducted a systematic literature review of qualitative studies to describe the experiences of patients with feeding tubes during treatment of head and neck cancer. They found, among other things, that conflicting views and mixed messages from medical staff can have a negative impact on the patient’s view of feeding tubes. However, some patients valued the opportunity to participate in the decision-making process. Patients experienced the transition from EN to ON as challenging. The study of Ehrsson et al. [51] included in the systematic review by Williams et al. [52] showed that patients recognize the value of tube feeding for survival and the importance of becoming involved in decision-making. Alongside these positive aspects, patients with head and neck cancer also reported negative experiences with feeding tubes, including irritation in the nose and throat, drooling, nausea and others [51]. No such data are available for ICU and post-ICU patients. In practice, it appears that ICU and post-ICU patients are often excluded from decisions about feeding tube removal, with their opinions given little consideration. If the basic biomedical ethical principle of autonomy according to Beauchamp and Childress [53] would be followed, the patient should also be asked about their opinions, views and wishes when making the decision to remove the feeding tubes after receiving the important information needed to take a decision (informed consent). Therefore, patients can only be involved in the decision-making if they are informed about their current nutritional situation, including their current oral requirements and realistic intakes, as part of shared decision-making [54]. Currently, the extent to which patients are included in these decisions remains unclear.

Studies showed that the removal of feeding tubes is often a primary goal of nurses and physicians in the ward and the decision to remove the tube is not made based on an estimation of eventual oral energy and protein intake. The process itself and the criteria for the decision of tube removal seems to be unclear. Further research is needed to comprehensively understand the decision-making process for feeding tube removal and to explore the experiences and perspectives of all participants, including patients.

### 4.6. Limitation and Strenghts of the Study

To the best of our knowledge, this scoping review is the first to address the topic of transition from EN to ON in post-ICU patients. By addressing this important issue, it highlights a problem and raises awareness of premature feeding tube removal, what we see as a strength of the review. Another strength lies in the rigorous methodological approach, including collaborative exchanges between the first and second authors throughout the review process. However, the socping review has certain limitations. The findings of the included studies are influenced by various factors, such as the country, the healthcare setting, the hospital organization, the area of practice of the healthcare professionals and the characteristics of the patient populations. These variables limit the generalizability of the results. Furthermore, the restriction to studies in English and German, as well as the scope of the databases searched, may have resulted in the omission of relevant studies.

## 5. Conclusions

In summary, ON remains a challenge for many patients even after ICU discharge due to persistent barriers. As a result, EN via feeding tubes are frequently necessary to meet energy and protein requirements and to prevent malnutrition after ICU discharge. The energy and protein requirements of post-ICU patients are not yet fully understood, and there is insufficient literature addressing this problem. Particularly, in the transition from EN to ON and its impact on energy and protein adequacy, many questions remain unanswered. Further research is needed to examine this transition process and its effects on the energy and protein adequacy and nutritional status of ICU and post-ICU patients. Additionally, more insights are required into the decision-making process surrounding feeding tube removal. This includes exploring the experiences, opinions, and perspectives of both healthcare professionals and patients to optimize the transition process and minimize the risk of premature feeding tube removal. Furthermore, the comparison of energy and protein adequacy across studies is hindered by inconsistencies in the methods used to calculate and report energy and protein requirements and intake. Further research is needed to define energy and protein needs of this population and standardize methodologies.

The scoping review provides an overview of the limited literature available on this topic and serves as a foundation for future research on the transition from enteral to oral nutrition in ICU and post-ICU patients. Given the current state of research, it is advisable to approach feeding tube removal with particular caution in this vulnerable patient group. A thorough assessment of the realistic potential for achieving adequate oral energy and protein intake should be conducted, ideally with the involvement of a clinical dietitian.

## Figures and Tables

**Table 1:** Potential benefits and harms of the transition process from enteral to oral nutrition in ICU and post-ICU patients.

Potential Benefits	
Physiological	Stimulates normal digestive process including salvation, gastric motility and enzyme secretion. Helps maintain gut mucosal integrity and microbiome health.
Quality of Life	Returns pleasure and social aspects of eating. Eliminates the potential discomfort of feeding tubes. Increases patient autonomy and self-esteem.
Clinical	Reduces the risk of potential tube-related complications (e.g., ulcers, dislodgement, clogging). May improve swallowing function through practice of oral intake.
Cost Reduction	Decreased need for medical supplies and feeding tube formula.
**Potential harms**	
Nutritional risks	Possible inadequate caloric and protein intake during and after transition. Risk of weight loss and malnutrition if oral intake is insufficient. Potential micronutrient gaps if diet variety is limited (e.g., consistency modified diet).
Safety concerns	Aspiration risk if swallowing function is impaired (dysphagia). Potential malnutrition.
Psychological	Anxiety and fear of eating (e.g., fear of failure, fear of choking). Loss of confidence and frustration if oral intake is difficult. Pressure to achieve nutritional goals by oral nutrition alone.

**Table 2:** Author, title, design, publication year and main findings regarding the research question of this scoping review.

Author	Title	Design	Year of Publication	Main Findings (Regarding Research Question of This Scoping Review)
Slingerland-Boot et al. [27]	Prospective Observational Cohort Study of Reached Protein and Energy Targets in General Wards during the Post-Intensive Care Period: The PROSPECT-I Study	Prospective observational single-center cohort study	2022	Decrease in median energy adequacy from 97.3% to 76% and in median protein adequacy from 91.5% to 75.4% of target values. Largest drop on day 1 after feeding tube removal. Median energy adequacy of 65% and a median protein adequacy of 60.6%. Lowest intake with ON only, best with EN support.
Chapple et al. [28]	Barriers to Nutrition Intervention for Patients with a Traumatic Brain Injury: Views and Attitudes of Medical and Nursing Practitioners in the Acute Care Setting.	Qualitative semi-structured interview study	2018	Feeding tube removal is a primary goal of nurses and physicians to receive them back to normal as soon as possible. Re-insertion of a feeding tube is experienced as setback and moving back. Weight loss was tolerated during recovery process. Nutrition seems not to be discussed often during ward rounds.
Merriweather et al. [29]	Nutritional Rehabilitation after ICU—Does It Happen: A Qualitative Interview and Observational Study	Qualitative grounded theory interview and observation study	2014	General ward ethos of removing feeding tubes as soon as possible after discharge from the ICU. Feeding tube removal happens often without formal assessment of energy and protein intake by a dietitian, and out of hours when no dietitian was present. The observation showed a minimal ON after feeding tube removal. Lack of nutritional knowledge about nutritional needs of post-ICU patients lead to prematurely tube removal.
Moisey et al. [30]	The Role of Nutrition Rehabilitation in the Recovery of Survivors of Critical Illness: Underrecognized and Underappreciated.	Narrative review	2022	Lack of knowledge resulting in removal of feeding tubes often directly after extubating or shortly after ICU discharge.
Zaher et al. [31]	Understanding Nursing Perspective towards Barriers to the Optimal Delivery of Enteral Nutrition in Intensive Care Settings	Cross-sectional survey study	2024	Feeding tubes are frequently removed and it is often unclear whether this is performed based on a physician’s prescription or not.
ApSimon et al. [24]	Enteral Nutrition on Discharge from Intensive Care and 30-Day Unplanned Readmission: An Exploratory, Retrospective Study of Association.	Single-center retrospective study	2024	In 71% feeding tubes were removed in the ward because of hospital practice and not because energy and protein needs were covered orally. The longer patients stayed in the ICU, the more likely it was that the feeding tube will remain in place even after transferring them to the ward. Authors recommend an education of staff about the importance of inadequate ON and feeding tube removal and that feeding tubes should be removed based on the individual’s ability to meet oral energy and protein requirements rather than institutional policy.

## Abbreviations

ICU	Intensive Care Unit
ON	Oral Nutrition
EN	Enteral Nutrition
PN	Parenteral Nutrition

## Appendix A

**Table A1:** Pubmed Search Strategy.

Date of Search	Database	Searching Terms
24 January 2025	Pubmed	enteral nutrition AND feeding tube AND nutritional status AND intensive care AND energy AND protein
24 January 2025	Pubmed	enteral nutrition AND feeding tube AND intensive care AND energy AND protein Filter: last 10 years
24 January 2025	Pubmed	Enteral nutrition AND feeding tube AND intensive care Filter: last 10 years
24 January 2025	Pubmed	“Critical Illness/therapy”[MeSH]) AND “Energy Intake”[MeSH]) AND “Intensive Care Units”[MeSH]) AND “Dietary Proteins”[MAJR] Filter: last 10 years
24 January 2025	Pubmed	“Critical Illness”[MeSH]) AND “Intensive Care Units”[MeSH]) AND “Nutrition Assessment”[MeSH]) Filter: last 10 years
27 January 2025	Pubmed	nutritional status AND nutritional assessment AND Intensive Care AND critical care AND feeding tube AND enteral nutrition Filter: last 10 years
27 January 2025	Pubmed	nurses AND physicians AND feeding tubes AND nutrition
12 February 2025	Pubmed	critical care AND nutrition AND qualitative research AND enteral
12 February 2025	Pubmed	clinical practice AND observation AND enteral AND feeding tube AND qualitative
18 February 2025	Pubmed	enteral nutrition AND feeding tube AND transition AND oral AND critical care
